# Head and Neck Cancer in Haiti: A Case Series from Hopital de L'Universite d'Etat d'Haiti

**DOI:** 10.1155/2018/9429287

**Published:** 2018-10-01

**Authors:** Maxwell P. Kligerman, Anahuma Alexandre, Patrick Jean-Gilles, David K. Walmer, Adam J. Gomez, Christina S. Kong, Mack L. Cheney, Murray A. Mittleman, Anna H. Messner

**Affiliations:** ^1^Department of Otolaryngology/Head & Neck Surgery, Stanford University, Stanford, CA, USA; ^2^Family Health Ministries, 501-C3, Durham, NC, USA; ^3^Department of Otorhinolaryngology, Hopital de L'Universite d'Etat d'Haiti, Port-au-Prince, Haiti; ^4^Duke University Global Health Institute, Durham, NC, USA; ^5^Department of Pathology, Stanford University, Stanford, CA, USA; ^6^Massachusetts Eye and Ear Infirmary, Harvard School of Medicine, Boston, MA, USA; ^7^Harvard T.H. Chan School of Public Health, Harvard University, Boston, MA, USA

## Abstract

This manuscript characterizes the demographics, presenting symptoms and risk factors of patients diagnosed with head and neck cancer at Hopital de L'Universite d'Etat d'Haiti (HUEH), Haiti's single largest healthcare facility. We conducted a prospective study of patients who presented to HUEH between January and March of 2016 with a lesion of the head or neck suspicious for cancer. All patients who met eligibility criteria received a biopsy, which was interpreted by a Haitian pathologist and when the specimen was available was confirmed by a team of pathologists from Stanford University. A total of 34 participants were identified. The biopsy-confirmed diagnoses were squamous cell carcinoma (n=7), benign (n=7), large cell lymphoma (n=2), ameloblastoma (n=2), pleomorphic adenoma (n=1), and adenocarcinoma (n=1). Fourteen patients were unavailable for biopsy. Patients with head and neck cancer had a mean age of 63.4 years, were majority male (62.5%), waited on average 10.9 months to seek medical attention, and most commonly presented with T-stage 3 or higher disease (87.5%). By characterizing patterns of head and neck cancer at HUEH we hope to facilitate efforts to improve early detection, diagnosis, and management of this important public health condition.

## 1. Introduction

The majority of global cancer deaths now occur in low and middle-income countries, where patients present at later stages of disease and have less access to curative treatment [1, 2]. Head and neck cancer in particular constitutes a significant proportion of the global cancer burden. As a whole, head and neck cancers are the fifth most common cancer and account for over 500,000 new cancer cases each year [3]. The global capacity to treat head and neck cancer, however, is unable to meet demand. Nearly two-thirds of head and neck cancers now occur in low and middle-income countries [4], yet the number of otolaryngologists in the world's poorest countries is as low as one per one-million [5].

In Haiti, the western hemisphere's poorest country, little is known regarding the burden of head and neck cancer or the prevalence of traditional risk factors. New research, however, has demonstrated that Haiti has the highest incidence of esophageal, stomach, and liver cancer in the Caribbean, and one of the highest rates of Human Papilloma Virus (HPV) induced cervical cancer in the world [6, 7]. Whether these trends might hold true for head and neck cancer is unclear. In this study we aimed to assess the epidemiology of head and neck cancer at Hopital de L'Universite d'Etat d'Haiti (HUEH), Haiti's largest healthcare facility [8]. These findings will add to the growing body of literature describing the global burden of head and neck cancer and facilitate efforts to improve early detection, diagnosis, and management in Haiti and other low and middle-income countries.

## 2. Methods

Participants were prospectively recruited at HUEH in Port-au-Prince, Haiti between January and March 2016. Patients 18 years of age or older with a clinically suspicious lesion of the oral cavity, oropharynx, hypopharynx, larynx, mandible, or neck that met at least three of the following four criteria were eligible to participate: (1) at least 2 cm in diameter, (2) increasing in size over time, (3) present for at least 30 days, and (4) nonpainful. Eligible participants provided written informed consent in Creole and completed a verbal interview modeled from the HOTSPOT survey that was administered by a Haitian physician [9]. Participants receiving a biopsy consented to have it examined first by a local pathologist in Port-au-Prince and then embedded in paraffin for transport back to the United States. A pathologist at Stanford University (CSK) evaluated hematoxylin and eosin (H&E) stained sections prepared at Stanford from the paraffin blocks. Immunohistochemical (IHC) stain for CD20 was performed in one case to adjudicate a discrepancy between carcinoma and lymphoma. P16 IHC was also performed to determine HPV status in cases of squamous cell carcinoma (SCC). A second pathologist at Stanford (AJG) performed a separate blinded review of cases where the Stanford diagnosis differed from the Haitian diagnosis. Final diagnosis was based on agreement between two pathologists.

Data manipulation and analysis was performed using STATA v.14.0 (StataCorp, College Station, TX). Differences in proportions were assessed using Fisher's Exact Test and differences in the average level of continuous measures were assessed with the Wilcoxan Rank-Sum Test. Statistical significance was determined by a two-sided type I error threshold of 0.05. Diagnoses of SCC and adenocarcinoma were grouped together as ‘cancer,' and diagnoses of benign, pleomorphic adenoma, and ameloblastoma were grouped together as ‘benign.' This study was approved by the Stanford University IRB, Family Health Ministries, 501-C3 IRB, and the HUEH ethics committee.

## 3. Results

### 3.1. Patient Presentation

Table 1 shows the demographic characteristics of the 34 study participants identified at HUEH in Port-au-Prince between January and March 2016. Patients with head and neck cancer had a mean age of 63.4 years, were majority male (62.5%), and waited on average 10.9 months to seek medical attention. Patients with benign conditions, on the other hand, were significantly younger than those with cancer and had a mean age of 42.4 years (p=0.02), were half male (50%), and waited on average 13.7 months to seek medical attention. The most common secondary symptoms amongst patients with head and neck cancer were weight loss (50%), dysphagia (25%), and fever (25%). Amongst all 34 participants (including both those with head and neck cancer and benign conditions) the most commonly affected anatomic subsites were the neck (n=10), oropharynx (n=9), oral cavity (n=8), larynx (n=5), and mandible (n=2). Two of the 34 participants (5.8%) had a history of HIV/AIDS.

### 3.2. Diagnoses

A total of 20 biopsies were performed: 8 were epithelial cancers (7 SCC and 1 adenocarcinoma), 10 were benign (7 general benign, 2 ameloblastoma, and 1 pleomorphic adenoma), and 2 were large cell lymphoma. Fourteen patients (41.2%) were unavailable for biopsy and remained undiagnosed.  Table 2 summarizes the clinical characteristics of the 8 patients found to have head and neck cancer. Patients with cancer (SCC or adenocarcinoma) presented with either T-stage 2 (n=1), T-stage 3 (n=4), or T-stage 4 (n=3) disease, with a mean lesion diameter of 5.9 cm (SE=4.3). Four of the eight patients found to have cancer also had nodal involvement. Sixteen of the 20 biopsies were transported back to the United States, including six of seven cases of SCC. All cases of SCC (including two in the oropharynx) were HPV negative. The panel of pathologists at Stanford revised the initial diagnosis for four patients: changing the diagnosis from SCC to adenocarcinoma (1), carcinoma in situ to large cell lymphoma (1), and severe dysplasia/carcinoma in situ to benign (2).

### 3.3. Risk Factors

**Figure 1:**
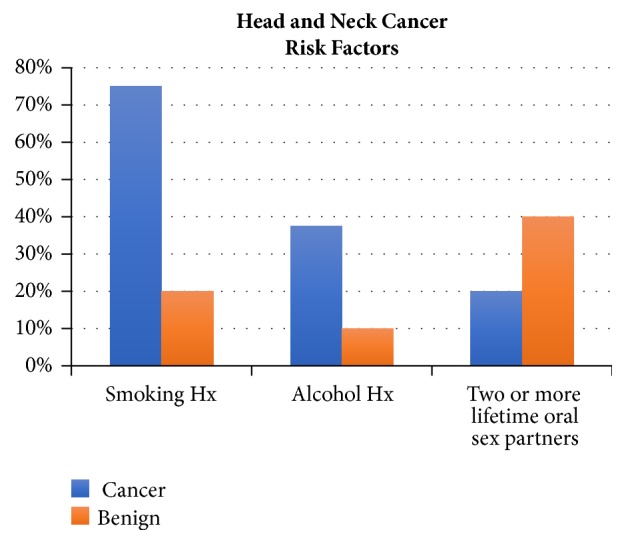
**Head and neck cancer risk factors**. Figure 1 demonstrates the major risk factors for those with and without a diagnosis of head and neck cancer.

Figure 1 shows the major risk factors for those with and without a diagnosis of head and neck cancer. Nearly one-quarter of all 34 participants had a history of cigarette smoking (n=8). Amongst those with cancer, 75% had a history of smoking (n=5) compared to 20% (n=2) of those with benign diagnoses (p=0.09). The mean number of pack-years amongst all smokers was 7 (SE 5.8). A history of habitual alcohol use (defined as at least one drink daily for 5 or more years) was present in 17.6% (n=6) of all participants. Amongst those with cancer, 37.5% (n=3) had a history of daily alcohol use compared to 10% (n=1) of those with benign diagnoses (p=0.21).

Sexual practices revealed no statistically significant differences between the head and neck cancer and benign groups. The number of vaginal sex partners for all 34 participants were as follows: 5.9% no lifetime vaginal sex partners (n=2), 47.1% one lifetime partner (n=16), 35.3% two to five lifetime partners (n=12), 8.8% six to ten lifetime partners (n=3), and 2.9% ten or greater lifetime partners (n=1). The number of lifetime oral sex partners for all 34 participants were as follows: 47.1% no lifetime oral sex partners (n=16), 26.5% one lifetime partner (n=9), 17.7% two to five lifetime partners (n=6), 5.9% six to ten lifetime partners (n=2), and 2.9% ten or greater lifetime partners (n=1).

## 4. Discussion

Head and neck cancer in low and middle-income countries remains a poorly understood public health concern. In this study we describe the epidemiology of head and neck cancer at HUEH, Haiti's largest healthcare facility, by prospectively identifying patients with head and neck cancer over a three-month period. During this time a total of eight patients with a new diagnosis of head and neck cancer were identified. Patients with head and neck cancer at HUEH had a mean age of 63.4 years, were majority male (62.5%), waited on average 10.9 months to seek medical attention, and most commonly presented with T-stage 3 or higher disease. Patient demographics are similar to those in high-income countries where males are two to four times more likely than females to develop head and neck cancer and the typical age of presentation is between 50 and 70 years [10]. The time to presentation and stage of presentation, however, at HUEH appear greater than those in high-income countries where the mean time to presentation is less than 6 months and where 40% of patients present with either T-stage I or T-stage II disease [11]. Considering that stage of presentation is one of the greatest predictors of head and neck cancer survival, future public health interventions may focus on facilitating hospital access and educating the general population about symptoms that require prompt hospital visitation.

Head and neck cancer risk factors at HUEH appear consistent with those in high-income countries. In high-income countries, nearly 75% of head and neck cancer (with the exception of oropharyngeal cancer) is attributable to tobacco or alcohol use [12]. In Haiti we find that 62.5% of patients with head and neck cancer had a history of either smoking or drinking. Rates of smoking in the general population in Haiti (10%) appear to be much lower than rates of smoking amongst patients with head and neck cancer at HUEH (62.5%) [13].

Whether HPV is a risk factor for oropharyngeal cancer in Haiti, however, remains unclear. In high-income countries, greater than 50% of oropharyngeal cancers are now associated with HPV [12], whereas rates of HPV induced oropharyngeal cancer in low and middle-income countries are as low as 17% [12]. In this study, just two cases of oropharyngeal SCC were identified. While both of these cases were negative for HPV, the small number of cases is insufficient to draw conclusions regarding overall trends at HUEH. It is interesting to note, however, that there are fewer lifetime sexual partners and lower rates of oral sex amongst patients at HUEH as compared to those in the United States, which is an important risk factor for HPV induced head and neck cancer. In the United States, for instance, nearly 90% of adults have had at least one lifetime oral sex partner compared to our findings of 53% in Haiti. We also find the median number of lifetime sexual partner in Haiti is just one compared to a median of 4 in the United States [14].

While determining long-term outcomes of patients with head and neck cancer at HUEH is beyond the scope of this study, it does appear that a relatively small percentage of these patients go on to receive surgical treatment. For the year of 2014, for instance, a total of 4 operations at HUEH were performed to treat head and neck cancer [15]; meanwhile in this three-month study we identified 8 new cases of head and neck cancer. It is also important to consider the surgical rate within the context of the large number of patients with suspected cancer diagnoses who were lost to follow-up for biopsies. This low surgical rate is likely due to the late stage of presentation, modest surgical infrastructure, prior hospital strikes, and the fact that just two otolaryngologist in Haiti have formal head and neck cancer surgical training [16]. In 2016, however, a new surgical facility dedicated specifically for the Department of Otolaryngology was built. Therefore, future efforts to promote patient follow-up and improve surgical capacity via physician training at HUEH may prove productive long-term strategies.

Another important finding of this study was the discrepancy in diagnoses between the Haitian and Stanford-based pathologists. We attribute this discrepancy to a lack of immunohistochemistry and other specialized staining techniques in Haiti, and absence of a mechanism for obtaining second opinions on cases where there is known high interobserver variability (e.g., diagnosis of epithelial dysplasia). Future efforts to bolster pathologic infrastructure may improve the accuracy of head and neck cancer diagnoses and in turn benefit all patients receiving biopsies.

This study has several limitations. First, the small study size results in low statistical power, making comparisons between patients with benign and cancerous diagnoses challenging. There was also a high rate of patients who were lost to follow-up, which may have distorted the types and rates of diagnoses. As with any survey that asks sensitive questions, it is also possible that respondents may have been reserved in their answers. Extrapolating these results to the general population of Haiti is challenging given the confounders that all participants had lesions suspicious of head and neck cancer and were recruited from the same hospital. One of the inclusion criteria (that the suspected lesion be at least 2 cm in diameter) may also have resulted in selection bias by predisposing enrolled patients to later stage disease. To the best knowledge of the authorship team, however, no potential participants were excluded for a lesion that was less than 2 cm in size.

It is also important to note that this study ended earlier than anticipated due to a medical staff strike at HUEH that started in March 2016 and resulted in the closure of the hospital for a four-month period [17, 18]. HUEH has since reopened but faced periodic closures due to strikes from nurses and hospital support staff [19]. The initial study protocol also called for collecting an oral brush specimen using the Hybrid Capture-2 High-Risk HPV DNA Assay (Digene Corporation, Gaithersburg, MD), however, laboratory closures in Port-au-Prince forced the discontinuation of this part of the study.

## 5. Conclusion

Head and neck cancer in low and middle-income countries like Haiti remains a poorly understood public health concern. We find that patients with head and neck cancer at HUEH, Haiti's largest healthcare facility, tend to present with late T-stage disease associated in large part with smoking and alcohol consumption. The rate of HPV induced head and neck cancer at HUEH remains unclear and is an important subject for future research. Efforts to improve head and neck cancer treatment at HUEH may focus on facilitating hospital access, bolstering surgical training and capacity, improving diagnostic and pathologic infrastructure, and educating the general public about cancer risk factors and symptoms that require prompt hospital presentation.

## Figures and Tables

**Table 1:** Patient presentation and demographics.

**Variable**	**Cancer (n=8)**	**Benign (n=10)**	**All (n=34)** **∗**	**P-Value** **∗** **∗**
Age (mean)	63.4	44.3	47.9	0.02
Male	5 (62.5%)	5 (50%)	15 (44.1%)	0.48
Literate	3 (37.5%)	8 (80%)	24 (70.6%)	0.09
Months to Presentation (mean)	10.9	13.7	11.43	0.77
Hx of HIV/AIDS	1 (12.5%)	0	2 (5.9%)	0.44
Symptoms:				
Fever	2 (25%)	1 (10%)	5 (14.7%)	0.56
Weight Loss	4 (50%)	5 (50%)	17 (50%)	0.68
Dysphagia	2 (25%)	5 (50%)	14 (41.2%)	0.28
Lesion Location:				
oral cavity	3 (37.5%)	3 (30%)	8 (23.5%)	0.56
oropharynx	3 (37.5%)	3 (30%)	9 (26.5%)	0.56
mandible	0	1 (10%)	2 (5.9%)	0.56
neck	1 (12.5%)	2 (20%)	10 (29.4%)	0.59
larynx	1 (12.5%)	1 (10%)	5 (14.7%)	0.71

*∗*All includes patients with cancer diagnoses, those with benign diagnoses, and those lost to follow who did not receive a biopsy.

*∗∗*p-value denotes comparisons between the cancer and benign groups.

**Table 2:** Head and neck cancer cases.

**Diagnosis**	**Gender**	**Age**	**Months to Present**	**Location**	**Diam. (cm)**	**Stage**	**HPV Status**
SCC	F	71	24	oropharynx	6	3	Negative
SCC	M	55	4	oropharynx	5	4	Negative
SCC	M	44	3	oral cavity	3	2	Negative
SCC	F	51	24	oral cavity	4.5	3	Negative
SCC	M	81	6	oral cavity	16	4	Negative
SCC	M	63	-	larynx	2	3	Negative
SCC	M	59	12	neck	6	4	-
Adeno	F	83	3	oropharynx	5	3	-

## Data Availability

The data used to support the findings of this study are available from the corresponding author upon request.

## References

[1] Jemal A., Bray F., Center M. M., Ferlay J., Ward E., Forman D. (2011). Global cancer statistics. *CA: A Cancer Journal for Clinicians*.

[2] Sharma K., Costas A., Damuse R. (2013). The haiti breast cancer initiative: Initial findings and analysis of barriers-to-care delaying patient presentation. *Journal of Oncology*.

[3] Torre L. A., Bray F., Siegel R. L., Ferlay J., Lortet-Tieulent J. (2015). Global cancer statistics, 2012. *CA: A Cancer Journal for Clinicians*.

[4] Warnakulasuriya S. (2009). Global epidemiology of oral and oropharyngeal cancer. *Oral Oncology*.

[5] Fagan J. J., Jacobs M. (2009). Survey of ENT services in Africa: Need for a comprehensive intervention. *Global Health Action*.

[6] Phillips A. A., Jacobson J. S., Magai C., Consedine N., Horowicz-Mehler N. C., Neugut A. I. (2007). Cancer incidence and mortality in the Caribbean. *Cancer Investigation*.

[7] Walmer D. K., Eder P. S., Bell L. (2013). Human Papillomavirus Prevalence in a Population of Women Living in Port-au-Prince and Leogane, Haiti. *PLoS ONE*.

[8] Tran T. M., Saint-Fort M., Jose M.-D. (2015). Estimation of Surgery Capacity in Haiti: Nationwide Survey of Hospitals. *World Journal of Surgery*.

[9] Anderson K. S., Gerber J. E., D'Souza G. (2015). Biologic predictors of serologic responses to HPV in oropharyngeal cancer: The HOTSPOT study. *Oral Oncology*.

[10] Lambert R., Sauvaget C., De Camargo Cancela M., Sankaranarayanan R. (2011). Epidemiology of cancer from the oral cavity and oropharynx. *European Journal of Gastroenterology & Hepatology*.

[11] Vernham G. A., Crowther J. A. (1994). Head and neck carcinoma – stage at presentation. *Clinical Otolaryngology and Allied Sciences*.

[12] Shield K. D., Ferlay J., Jemal A. (2016). The global incidence of lip, oral cavity, and pharyngeal cancers by subsite in 2012. *CA: A Cancer Journal for Clinicians*.

[13] WHO (2015). *WHO Report on the Global Tobacco Epidemic, 2015*.

[14] Chandra A., Mosher W. D., Copen C., Sionean C. (2011). Sexual behavior, sexual attraction, and sexual identity in the United States: Data from the 2006-2008 national survey of family growth. *National Health Statistics Reports*.

[15] Kligerman M. P., Alexandre A., Jean-Gilles P., Walmer D., Cheney M., Messner A. H. (2017). Otorhinolaryngology/Head and Neck Surgery in a low income country: The Haitian experience. *International Journal of Pediatric Otorhinolaryngology*.

[16] Jean-Gilles P. In: M. K, ed2017

[17] Cornish A. (2016). *Public Hospitals In Haiti Struggle To Stay Open As Doctor Strike Drags On*.

[18] Dyer O. (2016). Haiti's health system collapsing under weight of doctors' strike. *British Medical Journal (Clinical Research Ed.)*.

[19] McFadden D. (2017). Staff strikes again shutter Haitis public hospitals. *Salon*.

